# LifeChair: A Conductive Fabric Sensor-Based Smart Cushion for Actively Shaping Sitting Posture

**DOI:** 10.3390/s18072261

**Published:** 2018-07-13

**Authors:** Karlos Ishac, Kenji Suzuki

**Affiliations:** School of Integrative and Global Majors, University of Tsukuba, 1-1-1 Tennodai, Tsukuba, Ibaraki 305-8573, Japan; kenji@ieee.org

**Keywords:** posture, pressure sensing, conductive fabric, haptic feedback, interfaces, cushion, classification

## Abstract

The LifeChair is a smart cushion that provides vibrotactile feedback by actively sensing and classifying sitting postures to encourage upright posture and reduce slouching. The key component of the LifeChair is our novel conductive fabric pressure sensing array. Fabric sensors have been explored in the past, but a full sensing solution for embedded real world use has not been proposed. We have designed our system with commercial use in mind, and as a result, it has a high focus on manufacturability, cost-effectiveness and adaptiveness. We demonstrate the performance of our fabric sensing system by installing it into the LifeChair and comparing its posture detection accuracy with our previous study that implemented a conventional flexible printed PCB-sensing system. In this study, it is shown that the LifeChair can detect all 11 postures across 20 participants with an improved average accuracy of 98.1%, and it demonstrates significantly lower variance when interfacing with different users. We also conduct a performance study with 10 participants to evaluate the effectiveness of the LifeChair device in improving upright posture and reducing slouching. Our performance study demonstrates that the LifeChair is effective in encouraging users to sit upright with an increase of 68.1% in time spent seated upright when vibrotactile feedback is activated.

## 1. Introduction

It is estimated that we spend more than half our waking day in a sedentary state, and it is negatively affecting our physical, mental and psychological health [1]. Prolonged sitting and slouching are becoming a serious issues of concern due to the rise of desk-type jobs and hand held device use. Slouching is detrimental to our health as it disturbs the natural curve of the spine, produces back and neck pain [2], restricts blood circulation and increases our risk of death [3]. It additionally causes stress, fatigue [4], unproductivity and poor mood [5,6]. Upright sitting has many benefits for human health in the prevention of injuries, improvement of mood and increase in work productivity [7,8,9]. The LifeChair is a smart cushion for the back of your chair that uses our novel pressure sensing system, vibrotactile feedback and posture classifier to actively train sitting posture. It does this by detecting poor posture and sending haptic alerts to encourage the user to sit upright [10]. By using unique vibration patterns we communicate to the user spatial information, such as how to correct their posture. The LifeChair system can detect 11 different sitting postures with an accuracy of 98.1% and has demonstrated effectiveness in increasing upright posture duration by 68.1%. The key component of the LifeChair is the design and implementation of the specially developed fabric pressure sensing array and our posture classification model.

Pressure sensors have been widely used in many interfaces in robotics as a form of input for understanding human behaviour. Many systems tend to use off the shelf components such as force sensitive resistors (FSR) and flexible printed circuit boards (PCBs). Lokavee et al. [11] uses the conventional FSR sensing array to detect sleeping behaviour among users. The system in [12] uses FSR sensors on a sitting mat for the bottom of the chair to monitor extended sitting time. FSR sensors tend to be expensive to manufacture in large quantities and lack customization. The plastic base of FSR sensors, especially in large sizes, produces an unnatural, restrictive interface which is difficult to fit onto complex objects, and, as reference [13] further points out, FSR sensors lack performance with changes of curvature, temperature and biological tissue compliance.

Fabric-based sensing has become especially popular with the increased interest in soft robotics and adaptive and wearable interfaces. However, most of these do not offer a full scalable pressure sensing solution and are often used as simple buttons, in small sizes and in combination with conventional flexible PCB sensors. Fabric sensors also suffer from cross talk more than other types of sensors [14]. In reference [15], two methods to eliminate crosstalk are proposed; however, the solutions either increase the processing time required for data analysis or require the addition of further circuitry, such as multiplexers and diodes, which add to the system complexity. Our system avoids cross talk through sensor isolation and the individual layering of fabrics. We also allow for high frequency sampling rates due to the simplicity of our circuitry and sensing system. The work presented in [16] uses a fabric sensor array for in-shoe plantar analysis. However the design itself is quite thick and cannot be considered to be embedded approach due to its size and lack of flexibility. In reference [17], a sensor array for gait analysis was proposed. Their approach is more suited for small size arrays and still contains components of rigid plastic sheets. As the size of the sensor array is increased, the rigidity due to the plastic sheeting layers and complexity of the circuitry will also increase, making it impractical for larger size applications. The work in [17] also requires careful, manual trimming and cutting of the array to ensure performance. This may restrict the customisability of the sensor array and limit the feasibility for real world implementations.

We designed and developed a conductive fabric based pressure sensing array that provides force sensitive output, can be used in large variables sizes and has demonstrated manufacturability and customizability. We propose this as a full fabric sensing array solution that can be tailored for specific real world applications. We have also reduced the cross talk phenomena via eletrical isolation and signal filtering. We demonstrate the usability of our pressure sensing system by detecting 11 different sitting postures with a 98.1% accuracy.

We designed the LifeChair for real world use, typically for office working environments. Even with ergonomic chairs and passive posture assisting devices, we may still adopt poor posture, continue to slouch and spend a prolonged amount of time sitting down. There is no active feedback reminding us to maintain good posture. The LifeChair provides active real time feedback to solve this problem while monitoring the user’s sitting posture and activity through pressure sensing. Our previous study [10] demonstrated effectiveness in encouraging upright posture. In the study presented in this manuscript, we improve upon our posture classification model by developing our own pressure sensing technology, implementing it into the LifeChair and conducting a user study to detect 11 different sitting postures. We also evaluate the differences in participants’ sitting postures when the LifeChair system is activated and prove its benefits in reducing slouching and imbalanced sitting. Our main contributions are as follows:

1. The development of a pressure sensing system for embedded implementation on simple and complex surfaces. It is comprised primarily of conductive fabrics which are designed and arranged based on human biomechanics. This sensing system focuses on manufacturability, customisation and adaptibility.
2. A smart cushion interface for the backrest of a chair which has not been thoroughly explored. The benefits of the LifeChair cushion interface allow it to be a more flexible, adaptive and portable approach for posture correction than previous methods which attach sensors directly onto the chair backrest, underneath the chair, on the user’s seat or on the user themselves.
3. A posture classification model which can detect up to 11 postures based on the time series input of data from the pressure sensing system. The model was designed and verified based on training data and experiments from our previous studies.
4. We have developed a dedicated and sophisticated LifeChair smartphone application, and as a result, extensive testing and improvements have been included to ensure it is accurate and robust enough for real world use. Previous works have mainly opted for restrictive wired connections [18].

### 1.1. Sensing Sitting Posture

Continuous posture tracking and correction covers many domains including the workplace, biomedical, personal fitness, occupancy monitoring and gaming. The expansion of this domain has lead to several mobile applications to monitor sitting and standing posture [19,20]. However, it is still difficult to realize continuous posture tracking and correction on the seat due to limited sensor capabilities on the smart phone which often result in inaccurate feedback. Another issue is that the haptic feedback provided by a phone is quite limited in providing useful spatial information to the user. We, therefore, propose a more informative and embodied interface.

Pressure sensing interfaces have demonstrated accuracy in the detection and tracking of posture in both blind and known studies [21]. Furthermore, haptic feedback has demonstrated effectiveness in generating sensory-motor responses in human posture [22,23]. Vibrotactile cues have also been successful in training upright sitting posture and it has been shown that when haptic feedback is disabled, subjects continue to sit upright [18]. However, the placement of sensors in the system is not a good indicator of upright posture, since the shoulders and lumbar sides are not accounted for and only two FSR are placed on the back of the chair. Predominantly, the sensors used in reference [18] were small in size and could not give an accurate estimation of true upright posture, as mentioned in their discussion. In our system, we accurately detect pressure at various posture critical locations on the user’s back, including the shoulders, side lumbar and along the spine.

### 1.2. Conductive Fabric Sensing Technology

The advantages of fabric-based pressure sensors include their customizability, adaptability, cost effectiveness and scalability for real world implementation. Due to the fabric nature of this sensing method, it has vast potential to be embedded in everyday objects that we interact with. Reference [24] used conductive fabric-based sensors on a chair to monitor conversational movements in a user’s sitting posture. With the trend towards virtual reality in recent times, many research studies have explored the development of e-skin technologies as wearable interfaces [25]. There have also been studies which have explored similar applications for the biosensing of vital signs [26]. The research in [27] developed a fabric-based keyboard sensor which allows for a more flexible and lightweight keyboard. Furthermore, the work in [28] used fabric-based sensing on a table to monitor a dining experience. These examples demonstrate the capability of fabric-based sensors to be used in everyday real world scenarios.

### 1.3. Posture Training Interfaces

There are many types of posture training devices, including (1) cushion types, (2) mobile types and (3) wearable types. In our research, we utilized a cushion type interface due to its non-invasive approach and functional capabilities. Other cushion type systems, such as Cushionware [29] and eCushion [30], have attempted to detect sitting posture by using a pressure sensing pillow that the user sits on. These systems tend to move around as the user is sitting and are very basic indicators of good posture as they can only scan for simple pressure equilibrium or pressure along the spine. This does not guarantee good posture, as the user may still be experiencing the common rounded shoulders posture from mobile phone and electronic device overuse [31] or the forward head posture [23] which are primary sources of sitting posture-based discomfort [32]. Wearable systems, such as Waiston [33], detect posture using a basic tilt sensor, yet this is not a good indicator of posture as it does not prevent rounded shoulders, the forward head problem and slouching to the sides. Our system employs a less invasive approach by using a cushion interface attached to the back of the chair, ensuring it will not move around as the user shifts their posture or transitions between sitting and standing.

### 1.4. Designing Vibrotactile Feedback

Effective vibrotactile feedback as a communication cue on a human’s back has been previously explored [34,35]. A challenging aspect is the size, location, power and distance between tactors. As outlined in this study [36], the ideal spacing for lower back haptic acuity is 36 mm to 63 mm between motors. In the design of our spatial haptic feedback system, we used previous literature [37] as a foundation but experienced that there are many factors that can effect the feedback quality. One factor in the tactor design was the material interface. In our case, the vibrotactile force was partially absorbed by the foam material of the cushion, and so, increased horizontal and vertical spacing between motors was required for distinguishable spatial feedback to the user. This study [38] explored the affective reaction of haptic feedback as a positive or negative cue. It was demonstrated that haptic feedback as a negative cue more effectively generated a feedback response from the user. In our system, we chose to implement vibrotactile feedback as a form of negative cue.

## 2. Materials and Methods

### 2.1. Pressure Sensing Surface: Design and Methodology

#### 2.1.1. Design Requirements

In order to develop a new type of pressure sensing system that is suitable for complex interfaces and daily human interaction, we outlined several design criteria. These criteria are mainly targetted at developing an adaptive sensing surface for various chair shapes and sizes; however, they may also be used for implementation in a vast amount of interfaces, especially those with healthcare applications. The developed pressure sensing system should

- primarily consist of conductive fabric for flexibility and adaptivness;
- be sensitive to a range of small to large human forces;
- be manufacturable and cost-effective for commercial implementation;
- have a biomechanics-based design to accommodate for various users;
- be comfortable and compliant for humans; and
- have consistency over large sensor diameters (10 cm).

#### 2.1.2. Materials Compositions

**Figure 1:**
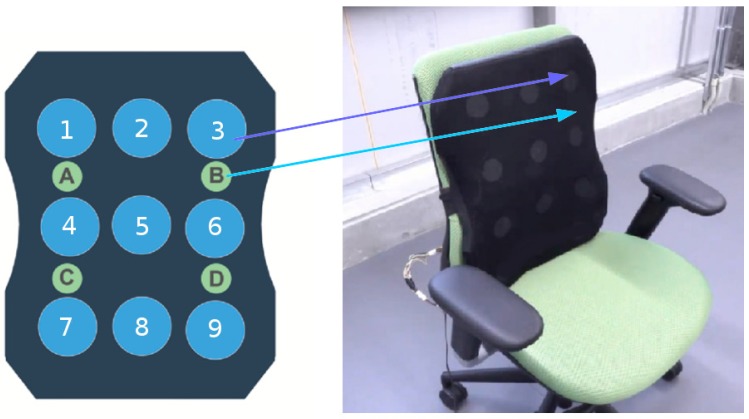
LifeChair pressure sensor arrangement (*i* = 1…9) and vibration motor locations (*j* = *A*…*D*).

**Figure 2:**
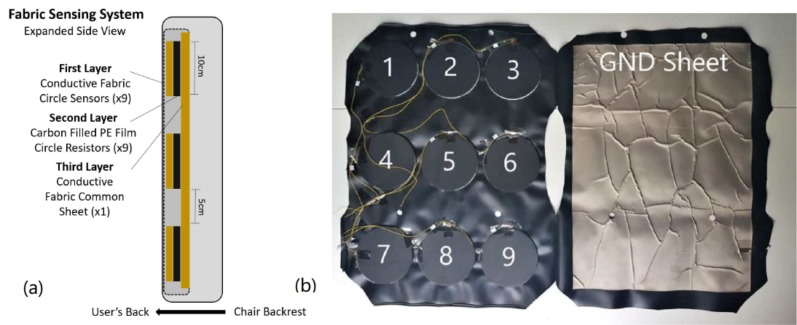
(**a**) Description of material layers in the proposed pressure sensing system; (**b**) fabric sensing system composed primarily of conductive fabrics (Ni, Cu and polyester) and conductive film. The left side shows the sensors with the conductive film visible; the fabric cloth is beneath the film. The right side shows the common fabric sheet.

Our sensing system uses conductive fabric as the primary component of the sensing mat to allow for adaptive and embedded pressure sensing. The dimensions and arrangement of the sensors and vibration motors are depicted in Figure 1. The final fabric sensors and materials are further detailed in Figure 2 and are composed of 3 layers. All materials described were acquired through factory machining, tooling and manufacturing based on our design requirements. The following description refers to the layer arrangement of the sensing system as if it were implemented into the LifeChair cushion and attached to the back side of a chair. The first layer is the layer closest to the user’s back.

1. The first layer is composed of single sided plain weaved conductive cloth and houses the individual nine circular sensors. The conductive fabric material used in this sensing mat is 0.08 mm thick and is a mixture of nickel, copper and polyester which is woven into the fabric and supported by an acrylic adhesive. The surface resistivity of the fabric is <0.05 Ω/sq. The conductive cloth itself is structured in 3 layers which contain the conductive cloth, conductive acrylic adhesive and releasing paper. Each fabric circle sensor is 10 cm in diameter based on our pilot studies and anthropomorphic design. Each sensor contains a piece of copper tape which has a wire soldered to it and is then connected to the respective pin on the PCB multiplexer. The length of each wire is dependent on the distance of each sensor to the PCB connection point. The conductive side faces towards the chair’s backside to allow it to interface with the conductive and resistive sensor layers.
2. The second layer is a 0.13 mm thick carbon filled polyethylene (PE) conductive film cut into 10 cm circles with <500 Ω/sq resistance. The conductive film is cut into 10 cm diameter circles to cover the surface area of the individual sensors but allow it to stay isolated from the neighbouring fabric sensors. Each conductive film circle behaves as a variable resistive layer for the respective circular sensor it is attached to. When force is applied, the resistance across the film drops and more current is allowed to pass through the conductive fabric layers.
3. The third layer (closest to backrest of the chair) is a sheet of rectangular conductive fabric that is composed of the same material as the individual sensors in the first layer. The fabric sheet is 38 cm in width and 50 cm in height and behaves as the common ground plane for the sensing system. It covers the surface area of all 9 circular sensors. There is a piece of copper tape attached to the edge of the conductive sheet closest to the PCB connection point. A wire is soldered to the copper tape and then connected to the GROUND pin on the PCB. The conductive side faces towards the user so that it can interface with the other conductive and resistive layers in the sensor array.

An anti-bacterial PVC cover, 0.52 mm thick, is used to cover the entire sensor array. Foam tape of 2.1 mm length is used to attach the top layer of sensors to the inside of the sensor mat, and fabric glue is used to attach the bottom layer of conductive fabric to the opposing inside layer of the cover.

#### 2.1.3. Sensors Circuit Design

The LifeChair operates on a Atmega328 processor which houses the sensing, motor driver, power and communication modules. The pressure sensing array functions mainly on a voltage divider circuit which is connected to a 16 channel multiplexer to interface with the 9 sensors. There are 9 analog outputs to the multiplexer which transmit the values to a serial interface to the smart phone application for real time visualisation. The layered sensors behave as a pressure sensitive resistor which is relative to force input on the sensor circle. The common sheet of conductive fabric is connected to the GND pin.

#### 2.1.4. Human Biomechanics-Based Sensor Arrangement

The pressure sensing system is designed primarily for the application of seated posture detection, and hence, is based on analysis of human upper body dimensions. In order to develop a device that maintains performance across users with varying body shapes and sizes, we chose to design the size of the sensors and the device based on the 5th percentile human adult US female (lower limit) and 95th percentile human adult US male (upper limit) seated shoulder width, height and hip width. Based on the referenced data [39] the seated shoulder width range is 37.5–50.5 cm, and the seated shoulder height is 50.5–64.6 cm. Furthermore, the hip width range is 31–40.5 cm. This range is considered in the minimum allowable distance between neighboring pressure sensors to ensure all sensors can be activated for different body types. Based on these measurements, we designed the LifeChair cushion to have a width of 40 cm, a height of 52 cm and a thickness of 3 cm. The circular pressure sensors were designed with a 10 cm diameter and are equally distributed along the center line with 11 cm spacing in the horizontal direction between centres and 17 cm spacing along the vertical direction between centres. Based on these dimensions, we also determined appropriate positions for the vibration motors based on our literature review and pilot studies. The positions of the vibration motors are depicted in Figure 1.

### 2.2. System Overview

#### 2.2.1. Outline of Components

**Figure 3:**
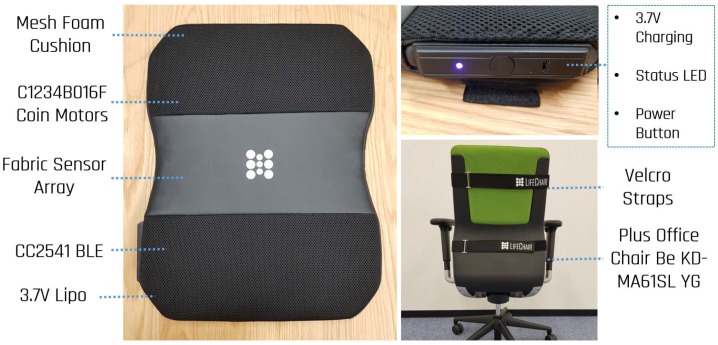
Overview of LifeChair components.

The main components of the LifeChair are depicted in Figure 3 and are the 55D foam cushion, the pressure sensing mat, 4 coin type C1234B016F vibration motors (KOTL, Zhejiang, China), the BLE module and a 3.7 V Li-po battery. All components are connected to a PCB encased and embedded in the lower left side of the LifeChair cushion. The encased panel has outlets for the RGB LED to notify the user of connection and power status, an ON/OFF power button and a micro-USB charging port. The chair used is the Plus Office Chair Be KD-MA61SL YG (Plus Corporation, Tokyo, Japan). The actuators used are all 3.0 V coin type motors, 12 mm in diameter and 3.4 mm in thickness and are located in each quadrant on the cushion equidistant from the center. With this approach, the user is given spatial awareness of their posture. This arrangement is unique to our system, as many alternative devices tend to vibrate the entire cushion or do not have a high resolution of back pressure to give accurate spatial feedback.

The LifeChair system is partnered with a smart phone application. The application communicates to the device using CC2541 BLE and allows the user to obtain direct visual representation of their pressure distribution, their classified posture and temporal postural statistics.

#### 2.2.2. Data Flow Protocol

**Figure 4:**
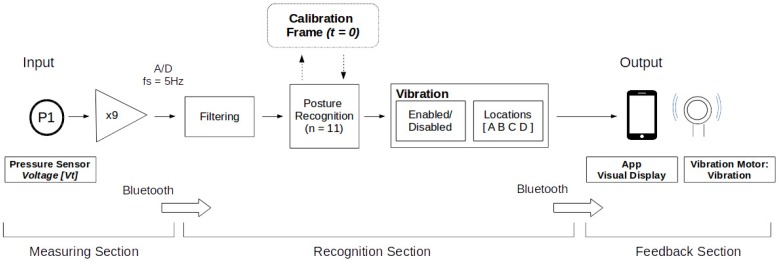
LifeChair data flow protocol.

The LifeChair uses bidirectional communication between the cushion device and the application. We employed a closed feedback loop where the sensor data is the input to the system and the vibrotactile feedback is the output. This process is detailed in Figure 4. The system first calibrates the LifeChair to the user’s upright posture through an application-guided calibration routine. Our application then scans the calibration snapshot for approximate balance in pressure distribution from the centerline and warns the user if an imbalanced calibration is detected. Once the calibration is verified, the sensor voltages are stored as a reference frame to compare deviations of pressure. The LifeChair then begins classifying posture in real time. The templates were constructed by a pilot study that measured sensor activation during different sitting conditions.

**Figure 5:**
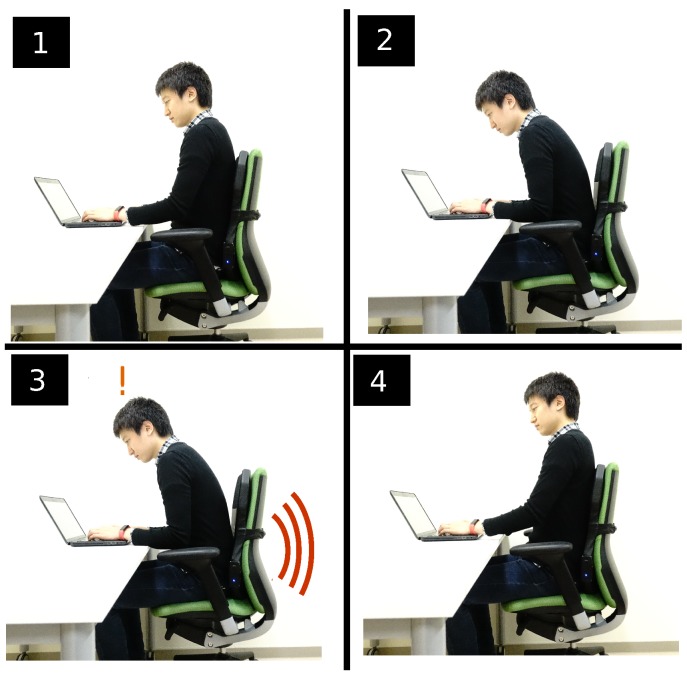
LifeChair system feedback loop: (**1**) Sensing sitting posture, (**2**) detecting slouching based on posture model, (**3**) sending vibrotactile feedback to the user to notify them of slouching, (**4**) user corrects their posture and vibration stops.

At first, in the measuring section, the LifeChair builds an array of the current pressure data across all 9 sensors. This data is then sent to the application via BLE. Once received, the pressure data is processed in the recognition section using our developed posture model from our previous study [10]. The algorithm then classifies the current seating posture. The detected posture is based on a comparison with the calibrated upright posture and uses an error threshold method. Based on the detected posture, a vibration sequence is sent back to the device which pulses until the user adjusts their posture. At this time, the classified posture is also displayed on the LifeChair application. This process is depicted in Figure 5.

#### 2.2.3. Posture Classification and Feedback

The sensors actively collect data at 200 ms intervals which is then matched to posture templates based on deviation of errors from the validated calibration reference array. These error regions are shown in Table 1.

The deviation errors are represented by ϵ for their respective sensors. We denote the calibrated force readings at each pressure sensor, $f_{i}$ ,where *i* is in reference to the sensor position shown in Figure 1 as 1…9. The array $V_{i}\left(t=0\right)$ refers to the voltage readings at all pressure sensors at the time of calibration given at t=0. The array $V_{i}\left(t\right)$ refers to the instantaneous voltage readings at time *t*. This is used to compute the error at each individual sensor’s location during active posture tracking.

(1) $$V_{i}\left(t\right)=\left[f_{1}\left(t\right)\ldots f_{9}\left(t\right)\right]\;.$$

(2) $$V_{i}\left(t=0\right)=\left[f_{1}\left(0\right)\ldots f_{9}\left(0\right)\right]\;.$$

The instantaneous error is denoted by $\epsilon {\left(t\right)}_{i}$ where ϵ is the computed deviation at time *t* at sensor *i*.

(3) $$\epsilon {\left(t\right)}_{i}={\left(V_{i}\left(t\right)-V_{i}\left(0\right)\right)}^{2}>\alpha \;$$

where α is a constant threshold determined from experimental pilot studies. If the computed error at each individual location is less than the threshold, α, then we assume that the user is seated in the upright position. If the deviation error at a particular sensor exceeds the threshold, then a reference posture is predicted based on a state lookup table. These states are denoted by $S_{p=1,\ldots ,9}$. The state look-up table also uses a variable, γ, to perform a priority-based search where it predicts the posture on a top-down look-up that ensures the state detected ranges from critical to specific. When the posture is predicted, it is displayed on the application, and the appropriate motors are pulsed until the user corrects their posture.

The training data was collected from 6 participants during a pilot study and then tested against in the experiments conducted in our previous publication [10]. The study conducted in [10] used 6 different subjects as a test for the training data. During this study, the classification accuracy was observed to be 94.1%. We tested the accuracy by instructing the user to sit in a random posture from the posture table and then observing if the detected posture matched the true posture of the user. Inaccuracies were counted if the detected posture was a different posture than the one currently occupied by the user or if there was an ‘unclassified’ case where the posture did not match any recorded pattern.

The training data was compiled and tested amongst several different threshold values to see which value could best fit the posture templates. The threshold used was a pressure deviation threshold which is relative to our upscaling of the pressure sensor voltages. It refers to the amount a user is allowed to shift from a particular pressure sensor (in our scaled signal range) before an ‘error’ at that particular sensor is deemed to have occurred. The coupled errors combine to give a classification of the current sitting posture.

#### 2.2.4. Haptic Function Design

In the LifeChair system, 4 vibration motors are utilized, each located in a quadrant on the cushion to approximately align with the human’s right shoulder, left shoulder, right lumbar and left lumbar. The positions of the motors are depicted in Figure 1. In order to ensure sensory motor response from the user, the feedback had to be designed with consideration to the vibration strength, frequency, size and position.

As previously mentioned, the objective of the LifeChair system is to use vibrotactile feedback as a medium for encouraging upright sitting posture and reducing slouching. The process is depicted in Figure 5. In stage 1, the LifeChair system actively senses and classifies the user’s sitting posture. In stage 2, the user begins to slouch as he uses his laptop, the LifeChair system detects this as an inappropriate posture based on our posture model. In stage 3, the user receives vibrotactile feedback based on the posture detected in stage 2. This vibration pulses until the user makes corrections to their posture. Finally, in stage 4 the user returns to the upright position and the vibration stops. In the case where the user is in an upright position or is not sitting down, no vibration is applied.

Based on our literature review and findings from a user feedback survey from our pilot study, we determined the optimal position, strength and frequency of the motors. The positions of the motors are depicted in Figure 1 and are represented by the letters A, B, C and D. The horizontal spacing between motors is 22 cm and the vertical spacing is 17 cm. This was determined from pilot studies that asked participants to distinguish between points of vibrotactile feedback on their back. The strength for pulsing the motors was in the range of 85 to 95% of the operating voltage. This was based on users steadily increasing the vibration strength until they felt the feedback was appropriate. The frequency of switching was also based on pilot experiments that trialled several different pulse width modulation (PWM) frequencies to explore what was the experimental value for alerting the user. Based on this, the PWM frequency for pulsing the vibration was found to be 4 Hz (250 ms).

## 3. Results and Discussion

### 3.1. Experiments

#### 3.1.1. Experimental Overview

The main apparatus for the experiment was the LifeChair cushion, as described in this work. We conducted three main experiments to test the functionality of the new fabric sensing system as well as the performance of the LifeChair device in classifying and improving sitting posture. The first experiment tested the pressure sensing system’s functionality. The second experiment used the newly developed pressure sensing system to detect 11 different sitting postures across 20 participants and compared the accuracy to our previous study [10]. The third experiment evaluated the effect of the LifeChair system in increasing time spent sitting upright and reducing slouching across 10 participants in a 2 h performance study.

#### 3.1.2. Experiment I: Pressure Sensor Array Feasibility Study

In this first experiment, we tested the feasibility, consistency, variance and force thresholds of the newly developed fabric sensing system. The aim was to develop a more adaptive system that could produce a higher posture detection accuracy than that of our previous system which used a flexible PCB system. In this study, we measured the resistance on each individual fabric sensor under various weights. The measured resistance was in reference to a common ground point on the fabric sheet, approximately where the sheet connected to the PCB. The sheet resistance of the conductive fabric used as the ground plane was less than 0.5 Ω. For this experiment, we used weights of 200 g, 1 kg, 2 kg, 5 kg and 10 kg. We repeated each trial for each sensor three times and computed the average resistance value.

#### 3.1.3. Experiment II: Posture Classification Accuracy

**Figure 6:**
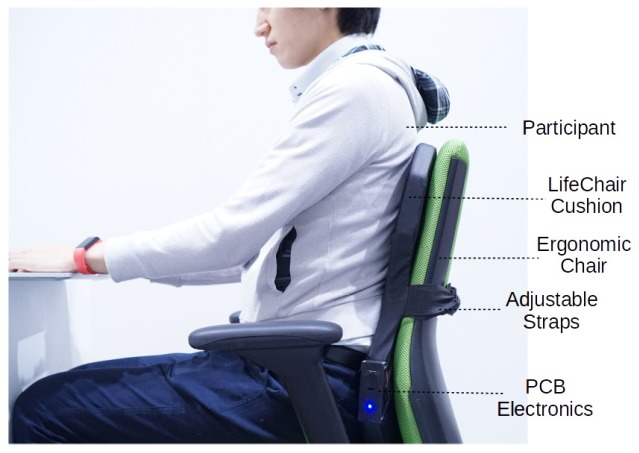
Experimental setup for classification experiments.

In this experiment, the participants sat on a chair equipped with the LifeChair system that had the newly developed pressure sensing system installed as shown in Figure 6. They were then asked to calibrate an upright position through a guided routine by the experimenter and the smart phone application. Once the upright position was verified by the visualization of sensors on the application, a snapshot of the pressure values was taken. An equilibrium check was performed and recalibration was suggested if necessary. The calibration was then used as the reference frame from which deviations in pressure were computed. Each participant was then instructed to sit in a random posture for three sets of 10 s until all postures had been registered, with a total of 33 trials. During this time, we compared the classified posture on the application to the true posture of the participant. The smart phone visualisation of the real time pressure distribution and posture classification was hidden from the participant during these experiments and was only visible during calibration. The classifier was based on training data collected during a pilot study that was tested and verified in our previous study [10].

The experiments were conducted with 20 healthy adult participants, 14 males and six females. All participants had no history of previous musculoskeletal or neurological disease, including no recent back or neck pain. All participants signed a consent form prior to participating in the study.

#### 3.1.4. Experiment III: Vibrotactile Feedback Effect on Posture

In this experiment, 10 healthy adult participants underwent a 2 h study where their posture was actively tracked. During this study, each user was instructed to work normally at their desk using a laptop/PC. All users were given a 10 min break between conditions. We observed the sitting posture of the user given the following conditions over the duration of one hour for each condition, as follows.

(A) No feedback condition: The participant did not receive haptic feedback in this mode but their posture was still tracked and recorded. The LifeChair system’s vibration motors were disabled but the pressure array still actively recorded the posture of the user. The pressure sensor data was timestamped and sent to the smart phone application where it was stored for analysis. This condition was used to observe the natural sitting behavior of the user when no feedback was given.

(B) Haptic feedback condition: The participant received spatial haptic feedback based on our posture-feedback model in Table 1. The feedback was given as a form of negative reinforcement indicating that posture should be corrected as well as the location at which correction was needed. The LifeChair actively recorded the pressure data of the user and communicated with the application to classify the posture and give appropriate feedback. The pressure sensor data was timestamped and stored on the smart phone application for analysis. This condition was used to observe how participants change their posture based on vibrotactile feedback as a form of negative reinforcement.

Condition (A) was run in the first hour of the performance experiment and condition (B) was run in the second hour. The conditions were not counter-balanced. We compared the differences in sitting behavior across both conditions to evaluate the effectiveness of vibrotactile feedback in encouraging upright posture and reducing slouching.

Before beginning this experiment, each user calibrated their upright posture which was checked and validated by the instructor, software criteria (balance checking) and the user themselves. We then checked each posture for valid detection by asking the participant to hold each of the 11 postures for 30 s and then validating it with the smart phone classification. Once an appropriate calibration had been detected, we began the study. We also checked the calibration in between conditions and at the end of the experiment to demonstrate the temporal accuracy and robustness of the system. It should be noted that there were no instances where a user had to recalibrate again at the start or during an experiment.

All participants signed a consent form prior to participating in the study.

### 3.2. Discussion

#### 3.2.1. Conductive Fabric Sensing System Evaluation

**Figure 7:**
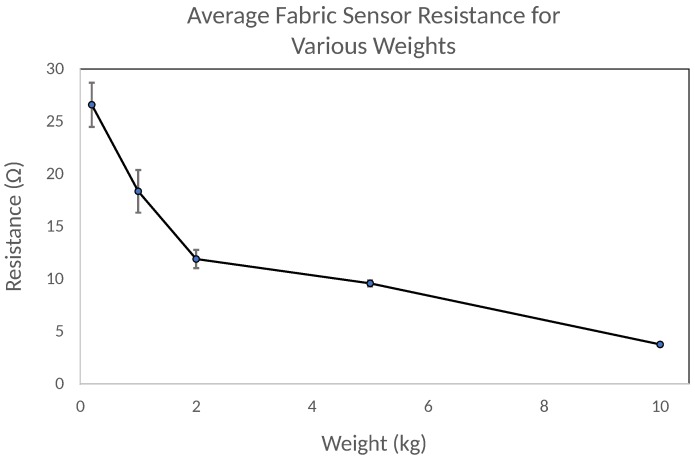
Average measured resistance for fabric sensors under various weights.

The results in Figure 7 show that across all of the individual pressure sensors, the resistance dropped when force was increased, demonstrating proper functionality. It also demonstrates the capability of the sensors to detect small forces of 200 g and large forces in excess of 10 kg; this shows the sensors feasibility for gathering sensitive postural movements, especially around the shoulders and lumbar regions. There was some variance detected under small pressures (<1 kg); the variance seemed to decrease between sensors as the force applied increased. Although this may have been filtered through a software calibration, it may indicate that the sensors are more suitable for applications that do not require a highly sensitive representation of small forces.

The fabric sensors also demonstrated more consistency between sensors compared to our previous flexible printed PCB system. In the previous system, sensors that were located further away from the PCB connection point had longer printed wires. As a result, there was variance in the resistivity between each sensor depending on its printed line length and thickness to the PCB. In this fabric-based system, we attached copper tape to each fabric sensor and then soldered a wire between the tape and the pcb connector. This helped to the eliminate variance in connector resistivity that we experienced with the flexible printed sensing system.

As we aimed to develop a customisable sensing system, we envisioned that the addition of an intermediate foam layer with through holes would increase the desirable force range for applications that require larger detectable pressures. The additional foam layer would behave as a physical resistor to allow the conductive layers to touch, This was an important consideration for our sensing system as our future works will explore the implementation of our fabric-based sensing into other interfaces that require larger force monitoring such as training correct balance, gait patterns and sleeping posture.

#### 3.2.2. Comparison of the Average Classification Accuracy for Sitting Posture Detection

Our previous study demonstrated a 94.1% posture detection accuracy using a flexible PCB sensing system. Although this value is considered high, there were several errors with the system when used with different participants. It was notably difficult to detect fine postural movements due to the physical bend resistance of the flexible PCB material fitting to the LifeChair and the chair’s shape. This made it limited for use in large sizes and as an interface with complex surfaces, essentially restricting its adaptiveness for embedded applications.

**Figure 8:**
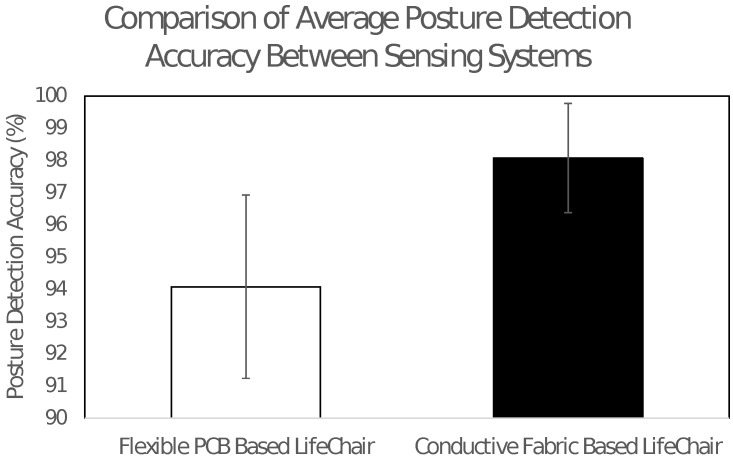
Comparison of average classification accuracies between sensing systems. The error bars are standard deviations (*F* = 3.198, *p* = 0.029).

In this research, we improved on these points by developing a fabric-based pressure sensing system that allowed more sensitivity and adaptiveness. As a result, in this study, we were able to classify 11 different sitting postures for all 20 participants with an average accuracy of 98.1%. Figure 8 shows a comparison of the average classification accuracies between the sensing systems and the standard deviations for each group. Our results show that the newly developed fabric-based LifeChair had a significantly lower standard deviation among all participants (*F* = 3.198, *p* = 0.029). Major postures, such as ’slouching forward’ or ’leaning back’ were more easily recognized with fewer classification errors. Two out of 20 participants experienced minor difficulties in posture classifications, especially around the lumbar region. These observations are consistent with our previous study and can be attributed to individual personal flexibility in shifting the lower back. This may require modification of the posture classification model parameters as opposed to the sensing system iteself. Misclassifications may also be attributed to a participant’s ability to hold a certain posture for an extended period of time without fatiguing. It was further observed that the fabric sensors allowed the LifeChair to bend more and adapt to the chairs shape due to the flexibility of the sensing system. This allowed for a better interface environment when classifying posture and, in turn, increased the sensitivity of detection.

Due to the compliance to postural movements and improved comfort of the sensing system, we were able to achieve better performance in both calibration and posture detection. The previous printed PCB method produced sound and a physically resistive sensation when pressed against. This made it non-ideal for human interaction and daily real world use. The fabric-based system allowed for a much more embedded approach and no notable sounds or physical resistance were observed during the posture classification experiments across all 20 participants.

These results verify the improved performance of the fabric-based system compared to a conventional flexible PCB approach. They also verify the robustness of the LifeChair system and highlight its usability in performance studies that may be conducted in real world office scenarios.

#### 3.2.3. Vibrotactile Feedback Effect on Posture

**Figure 9:**
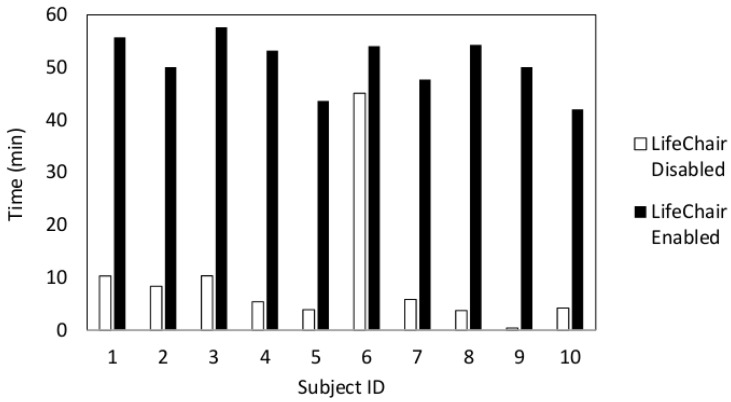
Vibrotactile feedback effect on posture: upright posture response for all 10 participants when LifeChair was disabled and enabled.

**Figure 10:**
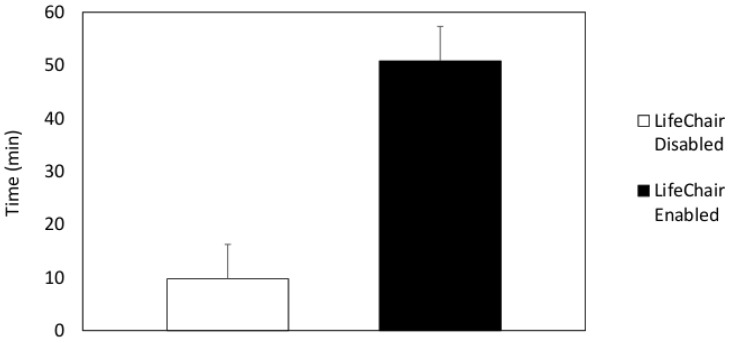
Average time spent upright for all 10 participants.

**Figure 11:**
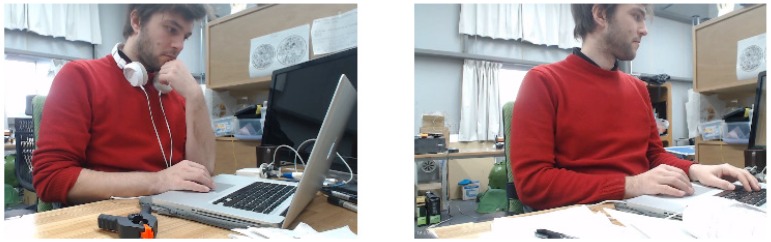
Live comparison of sitting postures during the vibrotactile response test when LifeChair was disabled (**left**) and when the LifeChair was enabled (**right**).

The percentage of time spent sitting upright for each user and the average value are displayed in Figure 9 and Figure 10, respectively. The results of the experiments in Figure 9 showed that all subjects experienced a higher percentage of time seated upright when the LifeChair system was enabled. The time spent upright per hour when the LifeChair was enabled increased from 56.7% from our previous system and studies [10] to 68.1% with the new LifeChair system and experiments presented in this study. This can be attributed to the improvement of the pressure sensor’s sensitivity and the implementation of a more human-centered design. Subject 6 may be considered as an outlier as they are a returning participant from a previous experiment which may have influenced their high duration of upright posture when the LifeChair was disabled. In further observations of the results for all participants, snapshots of the recordings (example Figure 11) showed that when the LifeChair was activated, the users would often adjust their workspaces to accommodate for better posture—in this manner they developed their own strategies based on the system’s feedback. Figure 11 also shows a snapshot of the experiment when vibration was disabled and when the user was augmented with the LifeChair system. The comparison clearly shows that the LifeChair system encouraged a broader posture, upright sitting and reduced slouching in the participant.

In our observations of the recordings, it seemed that the users responded mainly to the vibration feeling on the back. This is due mainly to some users actively responding while wearing earphones and the auditory signal from the tactors being not so noticeable. In fact, we tuned the vibration to specifically not disturb nearby people, as this device is envisioned to be used in an office, home or academic workspace.

**Figure 12:**
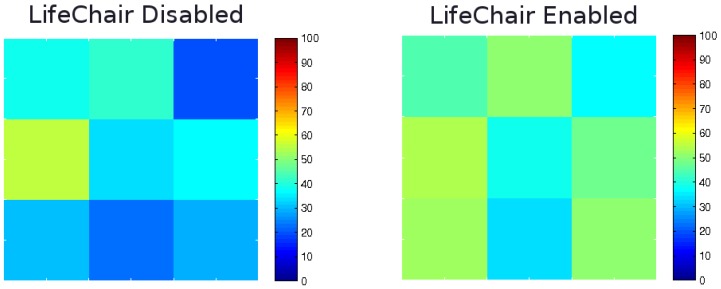
Heatmap of average pressure sensor activation per hour for all 10 participants when the LifeChair was disabled (**left**) and enabled (**right**).

Figure 12 shows a heat map of each pressure sensor’s activation, averaged across 10 users for a duration of one hour. The heat map shows a clear problem with the average user’s posture when not using the LifeChair system. We can observe that there is very little pressure in the right shoulder region, little lumbar contact and an overall uneven distribution of posture. Furthermore, we can observe the highest pressure zone being on the central left side. This is due to most people slouching to the left as a result of being right handed and having right hand-oriented workspaces. In the case where the LifeChair system was enabled, we can observe that the right shoulder problem was corrected, and as a result, the high pressure zone in the central left region reduced. Contact in the lumbar zone also increased and a much more equal distribution of overall pressure was achieved. Based on this, the LifeChair system is effective in reducing slouching, increasing lumbar contact and guiding the user to a more equally distributed healthy posture.

#### 3.2.4. System Evaluation

The combined results demonstrate that the LifeChair system was successful in detecting sitting posture across various participants with high accuracy and sensitivity. The developed fabric sensing system demonstrated higher accuracy, sensitivity and adaptiveness in detecting sitting postures when compared to our previous study and sensing method.

The customizability of the fabric sensing system is highlighted in the methodology section and further demonstrated in the results from Experiment I. Fabric sensors can easily be trimmed to suit different applications. Prior to this research, fabric sensors were explored as experimental components, often being used as a variable resistor in combination with conventional flexible PCB or FSR sensors. This is not a fabric sensing approach and is not feasible for truly embedded applications that require adaptiveness to complex shapes, such as the human body or ergonomic chairs. Our system has demonstrated adaptiveness to complex interfaces and embedded functionality and has demonstrated positive feedback in terms of function and comfort. In the future, we plan to further improve the customizability and adaptiveness of the system by replacing the copper wires with conductive thread materials [40].

The cost effectiveness criteria of the fabric sensing system was also achieved as the overall bill of materials to produce a single sensing mat with our design was slightly cheaper than producing a flexible PCB sensing mat of the same size and design. The main expense of the fabric sensing system is the human labour cost to manually solder each wire, whereas a flexible PCB has the wires directly printed on it. For a small sensing array of nine sensors, the difference is insignificant; however, this may become an issue when manufacturing larger sensor mats that require higher resolutions of sensing. We are currently exploring solutions to this as we intend to implement our system in other interfaces for the purposes of enhancing user mindfulness.

The increase in posture detection allows for a more robust foundation in computing the appropriate vibrotactile feedback to alert the user how they should correct their posture. With the increase in posture classification accuracy as a result of the fabric sensing system presented in this work, the LifeChair can be used in more dynamic posture sensing environments. The criteria outlined in our methodology section were achieved to a satisfactory level which, in turn, allowed us to classify 11 different sitting postures with a 98.1% accuracy across 20 participants while proving to be a responsive, comfortable and adaptive system.

The LifeChair performance experiments demonstrated that vibrotactile feedback is effective in training users to sit upright with an increase of 68.1% of time spent seated upright when vibration was enabled. Overall, all participants experienced an increase in time spent seated upright when the LifeChair was activated. Based on the results from the experiments, the use of vibrotactile feedback as a form of negative reinforcement for training upright posture is effective with our unique smart cushion interface. User feedback from the survey further praised the effectiveness of the LifeChair system in improving sitting posture.

#### 3.2.5. System Limitations

A notable limitation is the scenario in which the user is sitting on the edge of the seat and may not make contact with the LifeChair system. In this case, the pressure sensors will not be able to detect the user and cannot provide posture classification or feedback. We are currently working on several strategies to resolve this issue which include an extension of cushion compartments to the bottom of the seat. Furthermore, the need for recalibration after extensive use of the LifeChair is a key item of investigation in future works, as the workspace scenario may change or the user may become fatigued. In terms of the sensing system developed, fabric sensors themselves require more human labour work than other types of sensors, because of the manual soldering or threading of wires to each individual sensor. In high resolutions of sensor arrays, this approach may not be feasible. We are currently working on a method to manufacture large fabric sensor arrays with our proposed design whilst reducing the manual labour load.

## 4. Conclusions

In this research, we developed a smart cushion interface, named LifeChair, for posture correction that implemented our novel conductive fabric pressure sensor array and successfully improved the sitting posture of all participants. As part of this research, we developed an adaptable, flexible, cost-effective and human compliant pressure sensing system for use especially in embedded complex interfaces, such as smart cushions and chairs. Our pressure sensors use a novel fabric-based layering method which is proposed as a full solution for manufacturing and real world use. The flexible nature of fabric-based sensors allowed us to design for anthropomorphic paramaters of varying human back sizes and variations in chair shapes, whereas off the shelf FSR sensors and flexible PCBs have proved to be limited in this regard. The classification experiment demonstrated that the new LifeChair installed with the fabric sensing system could accurately classify 98.1% of all postures across 20 participants with significantly less variance than a flexible printed PCB system. After improving the system based on our initial findings [10], the performance study showed further improvement in posture correction by demonstrating a 68.1% increase in time spent seated upright when using the LifeChair which, in turn, reduced the overall time spent slouching. The LifeChair system was received positively and demonstrated overall effectiveness in improving sitting posture. In the future, we aim to implement our pressure sensing system into other healthcare interfaces for the purpose of empowering users’ mindfulness and augmenting training experiences.

## 5. Patents

The research presented in this article holds a certified patent with several claims covering both the LifeChair system and the pressure sensing technology.

## Figures and Tables

**Table 1:** Posture Classification and Haptic Feedback Model.

γ	$S_{p}$	ϵ	Vibration Pattern	Action Suggested
1	No User	$\Sigma_{i}^{n=9}\epsilon_{i}>n\alpha$	No Activation	Sit Down
2	Sitting Upright	$\Sigma_{i}^{n=9}\epsilon_{i}<\alpha$	No Activation	Maintain Upright Posture
3	Upper Back No Contact	$\epsilon_{1},\epsilon_{2},\epsilon_{3}>\alpha$	C and D	Sit Back in Upright Posture
3	Lower Back No Contact	$\epsilon_{7},\epsilon_{8},\epsilon_{9}>\alpha$	A and B	Pull back Lower Back
4	Slouching Forward	$\epsilon_{2},\epsilon_{5}>\alpha$	C and D	Sit Back in Upright Posture
5	User Slouching Right	$\epsilon_{3},\epsilon_{6},\epsilon_{9}>\alpha$	A and C	Pull back left side of body
5	User Slouching Left	$\epsilon_{1},\epsilon_{4},\epsilon_{7}>\alpha$	B and D	Pull back right side of body
6	Right Shoulder No Contact	$\epsilon_{1}>\alpha$	B	Pull back right shoulder
6	Left Shoulder No Contact	$\epsilon_{3}>\alpha$	A	Pull back left shoulder
6	Right Lumbar No Contact	$\epsilon_{7}>\alpha$	D	Pull back right lumbar
6	Left Lumbar No Contact	$\epsilon_{9}>\alpha$	C	Pull back left lumbar
